# Socioeconomic inequalities in the risk of suicide attempts among sexual minority adolescents: Findings from the UK's Millennium Cohort Study

**DOI:** 10.1016/j.lanepe.2022.100570

**Published:** 2022-12-26

**Authors:** James White, Sophie Borgia, David H. Rehkopf

**Affiliations:** a Centre for Trials Research, School of Medicine, Cardiff University, Cardiff, UK; b DECIPHer, School of Social Sciences, Cardiff University, Cardiff, UK; c Department of Epidemiology and Population Health, Department of Medicine, Department of Sociology, Center for Population Health Sciences, Stanford University, Stanford, USA

**Keywords:** Suicide attempt, Socioeconomic factors, Sexual minorities, Intersectionality

## Abstract

**Background:**

Both sexual minority and socioeconomically deprived young people are at an increased risk of making a suicide attempt. Intersectionality theory predicts these risk factors will interact synergistically to create unique vulnerabilities. We investigated the risk of suicide attempts in sexual minority socioeconomically deprived young people in a contemporary national cohort.

**Methods:**

The Millennium Cohort Study (MCS) is a birth cohort study in the UK following children born 2000–2002. Children in the MCS have been followed up over seven sweeps to date at ages 9 months, 3, 5, 7, 11, 14 and 17 years. The relative risk (RR) of self-reported suicide attempts at 17 years by sexual minority status and parental unemployment was estimated using multivariable log-binomial regression. Additive interaction, representing the synergistic effect, was estimated using the relative excess risk due to interaction (RERI).

**Findings:**

Between January, 2018 and March, 2019, 10,247 adolescents provided their sexuality and parents their employment status. 758 (7.4%) of 10,247 adolescents had made a suicide attempt. Relative to heterosexual young people living with no unemployed parents, the RR for sexual minorities living with no unemployed parents/carers was 2.93 (95% CI 2.26–3.79), one unemployed was 4.46 (95% CI 2.94–6.77), and two was 6.35 (95% CI 3.62–11.14). There was evidence of a positive additive interaction. The RERI for having one unemployed parent was 1.08 (95% CI −0.54 to 2.69) and two was 3.10 (95% CI −1.58 to 7.78). Sensitivity analyses using housing tenure and in a sample with no missing data generated comparable results.

**Interpretation:**

To our knowledge, this is the first evidence that socioeconomically deprived sexual minority adolescents are uniquely vulnerable to making a suicide attempt. Health and educational practitioners need to be aware of the increased risk of suicide attempts in socioeconomically deprived sexual minority adolescents.

**Funding:**

10.13039/501100000269Economic and Social Research Council (ESRC).

**Research in context**

**Evidence before this study**

We searched PubMed from inception to October 6, 2022, using the MeSH terms “sexual and gender minorities”, “socioeconomic factors”, and “suicide attempt”, with no language restrictions. We found population-based studies mainly based in the USA and Canada that focused on suicide attempts in either sexual minority or socioeconomically deprived youth. An additional search via Google Scholar identified studies on risk factors for suicide attempts. One study in the USA reported harassment of lesbian, gay, bisexual and transgender (LGBT) high school students was more common in districts with higher compared to lower levels of poverty. Another cross-sectional study of gay men reported those with lower incomes reported less social support than men with higher incomes. We did not identify any research that investigated suicide attempts at the interaction between sexuality and socioeconomic deprivation.

**Added value of this study**

By using data from a contemporary population-based UK cohort, we estimated the risk of suicide attempt was elevated more than would be expected in sexual minority socioeconomically deprived young people. This sample, born between 2000 and 2002, will have grown up in a society with more socially progressive attitudes and legal protections for sexual minorities in their childhood compared with previous generations. Despite this, sexual minority young people were many times more likely to report a suicide attempt than heterosexual people. Of the sexual minority adolescents, 32.6% with two unemployed parents/carers had made a suicide attempt, compared to 4.5% of heterosexuals living with no unemployed parents/carers. There was evidence of an additive interaction between sexual minority group status and the number of unemployed parents/carers young people lived with indicating a synergistic effect. To our knowledge, this is the first study that provides nationally generalisable estimates of the risk of suicide attempts in socioeconomically deprived sexual minority young people.

**Implications of all the available evidence**

This study provides evidence on the role of sexuality and socioeconomic deprivation in suicide attempts reported by the current generation of young people. Suicide attempt is the strongest predictor for future suicide. Policies, organizational practices, and universal school-based interventions that target risk factors (eg, improving equality and diversity training, reducing bullying) and preventing mental health problems in socioeconomically deprived sexual minority young people might be effective in supporting these young people and reducing the prevalence of suicide attempts.

## Introduction

Suicide is one of the leading causes of death in young people aged 25 years and under. In 2019, the Global Burden of Disease Study reported that suicide was ranked among the top five causes of mortality worldwide in 10–24 year olds after excluding central, eastern, western, and southern sub-Saharan Africa.^1^ Investigation into the association between socioeconomic deprivation and suicide has a history going back to the 19th century.^2^ Systematic reviews have shown area-level deprivation and income is associated with suicide rates,^3^ and people with a lower income, limited education, or a less prestigious occupation have around double the risk of suicide.^4^ Sexual minority (who identify as lesbian, gay, bisexual, or queer) young people also have higher rates of suicide attempts than their heterosexual peers. A systematic review of 35 studies found sexual minority people aged 12–20 years reported two to four times more suicide attempts than same aged heterosexual comparators.^5^ Relatively little is, however, known about whether the risks of socioeconomic deprivation and sexual identity interact synergistically to increase the risk of a suicide attempt.

Numerous theories have been invoked to explain these associations. Socioeconomic disadvantage has repeatedly been shown to be associated with mental illness, one of the strongest risk factors for suicide.^6^ The candidate mechanisms underlying the link between socioeconomic disadvantage and mental illness include chronic stress induced by exposure to negative life events including: financial hardship,^7^ parental illness,^8^ injury and death,^2^ violence,^9^ and shame.^10^ Minority stress theory suggests that sexual minority young people, due to their stigmatized identity,^11^ are exposed to more stressors than their heterosexual peers, such as bullying,^12^ a lack of social support,^13^ and stress related to the concealment or management of their identity,^14^ and as a result are at increased risk of making a suicide attempt.^15^^,^^16^

According to the theory of intersectionality,^17^ the effects of identification as a sexual minority and socioeconomic disadvantage may interact to create unique vulnerabilities that reflect the combined experiences of stress and marginalisation at the nexus of these two exposures.^18^ Although most commonly applied to investigate interactions between ethnic and other minority group identities, socioeconomic disadvantage has been a core dimension of investigation in intersectional inquiry from the outset.^17^ In statistical terms, the interaction between socioeconomic disadvantage and sexual minority status can be either additive or multiplicative, with the former representing the synergistic effect (i.e., the effects of both exposures on the risk difference scale exceeding the sum of effects), and later the sum of effects. Intersectionality theory,^17^ and the closely related theory of multiple jeopardy,^19^ implies a positive additive interaction between socioeconomic disadvantage and sexual minority status created by the compounding effects of both economic hardship and homophobia. We have, however, been unable to find any previous test of intersectionality theory on the risk of suicide attempts.

To address this research gap, we used data from a contemporary national birth cohort, to test a hypothesis that suicide attempts would be elevated in young people who are both socioeconomically deprived and a sexual minority.

## Methods

### Study design and participants

The Millennium Cohort Study (MCS) is a birth cohort study in the United Kingdom (UK) set up to follow the lives of children born at the turn of the new century.^20^ In total 19,519 children were recruited in 2000–02 and have been followed over seven sweeps to date at ages 9 months, 3, 5, 7, 11, 14 and 17 years. For information regarding sampling and survey design of the MCS see https://cls.ucl.ac.uk/cls-studies/millennium-cohort-study/.

We used data gathered at 9 months, 3, 14 and 17 years. In each wave, an interview is carried out with the main parent (normally the mother), resident partners, and, at ages 7, 11, 14 and 17, the cohort member. The outcome and exposures in the analysis were assessed when cohort members were 17 years of age. Data were collected for the sweep at 17 years of age between January 2018 and March 2019. Ethnicity reported by young people at 14 was used as it was not assessed at 17 years of age. In the sweep when cohort members were 17 years old, 14,496 families were invited to participate. Of this number, 10,625 (73.3%) families and 10,345 (71.4%) adolescents were successfully interviewed.

Ethics approval for the age 17 sweep was obtained from the National Research Ethics Service Research Ethics Committee (REC) North East–York (REC ref: 17/NE/0341). We adhered to the guidelines for STrengthening the Reporting of OBservational studies in Epidemiology (STROBE) in the reporting in this manuscript.^21^

### Measures

Attempted suicide was reported in response to the question, ‘Have you ever hurt yourself on purpose in an attempt to end your life?‘.

Sexual identity was self-reported with the response options of ‘completely heterosexual/straight’, ‘mainly heterosexual/straight’, ‘bisexual’, ‘mainly gay or lesbian’, ‘completely gay or lesbian’, ‘other’, ‘do not know’ and ‘preferred not to say’. The prevalence of these identities was, completely heterosexual/straight (76.8%), mainly heterosexual/straight (10.7%), bisexual (6.3%), mainly gay or lesbian (0.9%), completely gay or lesbian (1.5%), other (1.4%) and missing (2.4%). There is strong evidence that adolescents identifying as mainly or not sure they are a heterosexual have increased risk of mental health problems compared with those indicating they are completely heterosexual.^22^, ^23^, ^24^ In line with this literature, participants reporting they were mainly heterosexual were categorised as bisexual. Sexual minority status was defined as identifying as anything other than completely heterosexual/straight.

Socioeconomic disadvantage was assessed using information on the number of unemployed parents/carers resident with cohort members and derived from responses from parent(s)/carer(s) on their current employment status. The response options were: ‘employment’, ‘self-employment’, ‘looking after family’, ‘waiting to start a job’, ‘looking for a job’, ‘sickness/disability’, ‘being on a government scheme’, ‘full-time education’, ‘retirement’, and, ‘not in paid work, but not sick, on a training scheme, studying, retired or looking after the family’. Parents/carers who responded they were in employment or self-employment were combined. Those in the remaining categories were categorised as being unemployed/not economically active. The sum of the number of parents/carers who reported being unemployed/not economically active was then calculated.

Covariates included gender identity, assessed using self-reports from participants at 17 years of age as well as reports provided by parents at the 9-month and 3-year assessments. If young people at 17 years identified with a gender that was: ‘other’, ‘androgenous (male and female)’, ‘gender fluid’, or ‘non-binary’ they were categorised as a gender minority. If there was disagreement between parent reported male or female gender identity at 9 months or 3 years with young people's self-reported identification as male or female at 17 years of age, these participants were also categorised as gender minority. Ethnicity was self-reported by cohort members and categorised into: white; mixed, Indian, Bangladeshi or Pakistani, Black or Black British, other ethnic groups. Substance use comprised lifetime smoking experimentation (those who had one puff of a cigarette), consumption of a whole alcoholic drink, and illicit drug use. Housing tenure was reported by parents/carers to the question, “Do you own or rent your home or have some other arrangement?”. The response options were: ‘Own outright’, ‘Own—mortgage/loan’, ‘Part rent/part mortgage (shared equity)’, ‘Rent from local authority’, ‘Rent from Housing Association’, ‘Rent privately’, ‘Living with parents', ‘Live rent free’, and ‘Other’. Respondents who either owned outright or by mortgage/loan were combined as were the remaining options into two categories, owner occupier vs. non-owner occupier.

### Statistical analysis

A detailed description of attrition in the cohort is provided elsewhere.^25^ Missing data per variable ranged from 2.3 to 32.1%. Participants who reported that they: ‘didn't know’, ‘preferred not to say’, or ‘did not want to provide’ their gender (n = 47), sexual (n = 51), or ethnic identity (n = 56) were removed from the sample. There were 6952 participants with no missing data which made up the complete data sample. The resulting imputed analytical sample had 10,247 participants.

Missing exposure, covariate and outcome data was addressed through multiple imputation using chained equations under the assumption that data were missing at random. The imputation prediction model included all variables, along with combined sample and attrition weights,^26^ and an indictor variable denoting if participants were the only cohort member in the household or not. Estimates were obtained by pooling results across 20 imputed data sets using the Rubin rules, and assessment of Monte Carlo errors suggested this was a suitable number of imputations.^27^

We used multivariable log-binomial regression to estimate relative risks (RRs) and 95% confidence intervals for the associations between parental unemployment, sexual minority status, and the interaction between parental unemployment and sexual minority status on the risk of suicide attempt. Multiplicative interaction was assessed by evaluating whether the confidence interval of the interaction term included one. A positive multiplicative interaction would indicate the relative risk for suicide among sexual minority compared to heterosexual young people differ within the strata of being resident with no, one or two unemployed parents, and vice versa. We assessed additive interaction by calculating the relative excess risk due to interaction (RERI). This estimates the magnitude by extent to which a risk for a ‘doubly exposed’ group (eg, sexual minority and both parents/carers unemployed) differs from that expected based on the additive effects of each exposure.^28^ In the presence of additive interaction, the confidence intervals for the RERI would not include zero. No adjustments were made of the estimates for the main effects of sexual minority status, parental unemployment, the additive or multiplicative interactions.

To examine the influence of missing data we re-ran the analysis on a complete data sample. We also conducted two additional sensitivity analyses. We re-ran the models using a variable representing having any parents/carers who were unemployed. We then repeated the analysis using housing tenure as an alternative indicator of study members exposure to socioeconomic disadvantage.

All estimates were weighted using sample and attrition weights and computed using Stata 17 (StataCorp, College Station, TX) and R (version 4.03). Subgroup sample sizes were estimated from imputed proportions, weighted, and rounded so may not sum to totals.

### Role of the funding source

The funder of the study had no role in study design, data collection, data analysis, data interpretation, or writing of the report.

## Results

Of the 10,247 participants, 2493 (24.4%) lived with one unemployed and 214 (2.1%) with two unemployed parents/carers. 2177 (21.2%) of 10,247 participants identified as a sexual minority (Table 1).

There were 758 of 10,247 (7.4%) participants that reported a suicide attempt. The percentage of suicide attempts was higher in sexual minorities than heterosexuals (331 [15.2%] of 2177 vs. 427 [5.3%] of 8070). Suicide attempts were least common in heterosexual young people who lived with no unemployed parents/carers (265 [4.5%] of 5892), and most common in sexual minority young people who lived with two unemployed parents/carers (15 [33.2%] of 45). Relative to heterosexual young people who lived with two employed parents/carers, the RR for suicide attempts among sexual minority young people who lived with no unemployed parents/carers was 2.92 (95% CI 2.22–3.86), one unemployed was 4.64 (95% CI 3.09–6.98), or two unemployed parents/carers was 7.41 (95% CI 4.08–13.43) (Tables 2 and 3). We did not find evidence of a multiplicative interaction between parental unemployment and sexual minority status, but there was some evidence of a positive interaction on the additive scale, as the RERI for having one unemployed parent was 1.08 (95% CI −0.54 to 2.69, *P* = 0.13) and two was 3.10 (95% CI −1.58 to 7.77, *P* = 0.15) (Tables 2 and 3).

**Table 1:** Cohort member characteristics by parent/carer unemployment (n = 10,247).

	No unemployed parents/carers		One unemployed parent/carer		Two unemployed parents/carers	
	Heterosexual	Sexual minority	Heterosexual	Sexual minority	Heterosexual	Sexual minority
	(5892; 57.5%)^a^	(1648; 16.1%)	(2009; 19.6%)^a^	(484; 4.7%)	(169; 1.7%)^a^	(45; 0.4%)
***Gender identity***^b^						
Male	52.7	32.5	52.4	31.7	58.5	34.7
Female	47.1	62.9	47.5	63.3	40.9	59.3
Minority	0.02	4.6	0.01	5.0	0.06	6.0
***Housing tenure***						
Owned	79.4	80.3	54.9	56.0	25.4	29.0
Rented	20.6	19.7	45.1	44.0	74.6	71.0
***Ethnicity***						
White	83.0	87.8	61.4	79.1	59.7	80.0
Mixed	4.4	5.5	5.0	5.7	5.4	2.9
Indian/Pakistani/Bangladeshi	7.3	3.5	25.1	9.4	21.2	8.9
Black or Black British	3.5	1.7	4.1	3.2	5.4	4.4
Other	1.8	1.5	4.4	2.6	8.3	3.8
***Sexual identity***						
Heterosexual	100.0	–	100.0	–	100.0	–
Bisexual	–	83.4	–	78.7	–	62.7
Gay	–	10.5	–	14.0	–	21.0
Other	–	6.1	–	7.3	–	16.3
***Substance use***						
Lifetime smoking experimentation						
Yes	58.0	52.0	66.6	54.3	67.1	65.1
No	42.0	48.0	33.4	45.7	32.9	34.9
Lifetime alcohol use						
Yes	82.9	88.6	61.5	78.9	56.3	60.4
No	17.1	11.4	38.5	21.1	43.7	39.6
Lifetime drug use						
Yes	28.9	38.0	21.4	34.7	19.5	26.1
No	71.1	62.0	78.6	65.3	80.5	73.9

a All numbers estimated from imputed and weighted proportions.

b Gender minorities comprised cohort members who identified as androgenous, gender fluid, non-binary, or other.

**Table 2:** Interaction between living with one unemployed parent and sexual minority status on the risk of suicide attempt (n = 10,033).

	No unemployed parents/carers	One unemployed parent/carer	RR (95% CI) for living with One Unemployed Parent/carer within strata of Sexual Orientation
	RR (95% CI)	RR (95% CI)	
Heterosexual	1.00	1.65 (1.09–2.48)	1.65 (1.09–2.48)
Sexual Minority	2.92 (2.22–3.86)	4.64 (3.09–6.98)	1.59 (1.03–2.44)
RR (95% CI) for Sexual Minority within strata of living with one Unemployed Parent/carer	2.92 (2.22–3.86)	2.82 (2.03–3.92)	

Measure of interaction on additive scale: RERI (95% CI) = 1.08 (−0.54 to 2.69), *P* = 0.13.

Measure of interaction on multiplicative scale: RRs (95% CI) = 0.96 (0.62–1.49), *P* = 0.83.

RR: relative risk. CI: confidence interval.

**Table 3:** Interaction between living with two unemployed parents and sexual minority status on the risk of suicide attempt (n = 7754).

	No unemployed parents/carers	Two unemployed parents/carers	RR (95% CI) for living with Two Unemployed Parents/carers within strata of Sexual Orientation
	RR (95% CI)	RR (95% CI)	
Heterosexual	1.0	2.34 (0.65–8.47)	2.34 (0.65–8.47)
Sexual Minority	2.92 (2.22–3.86)	7.41 (4.08–13.43)	2.53 (1.23–5.23)
RR (95% CI) for Sexual Minority within strata of living with Two Unemployed Parents/carers	2.92 (2.22–3.86)	3.16 (0.90–11.17)	

Measure of interaction on additive scale: RERI (95% CI) = 3.10 (−1.58–7.78), *P* = 0.15.

Measure of interaction on multiplicative scale: RRs (95% CI) = 0.08 (−1.19, −1.34), *P* = 0.86.

RR: relative risk. CI: confidence interval.

In the sensitivity analyses, the RERI for the interaction between sexual minority status and living with any unemployed parents was 3.85 (95% CI 0.43–7.27, *P* = 0.03, Table S1) and living in a rented compared to owned house was 2.41 (95% CI 1.12–3.69, *P* < 0.001, Table S2). In the data sets in which there were no missing data, the CIs for estimates overlapped with those from the main results using imputed data (Tables S3 and S4).

## Discussion

Using data from a contemporary national cohort, we observed a 2 times higher risk of suicide attempt in sexual minority young people who lived with no unemployed parents, 4 times higher in people living with one unemployed, and 7 times higher risk in young people living with two unemployed parents/carers. We found evidence that socioeconomically deprived sexual minority young people had a risk of suicide that was greater than that expected based on the effects of being a sexual minority and socioeconomically deprived.

In agreement with previous research, we found evidence that exposure to socioeconomic disadvantage^29^ and sexual minority group membership^5^ were associated with an increased risk for suicide attempts. There has, however, been almost no research into the risk of suicide attempts for people at the nexus of socioeconomic disadvantage and sexual minority status. There have been a few studies on mechanisms that may explain the elevated risk at this intersection. One cross-sectional study in Columbia (United States) with lesbian, gay, bisexual and transgender (LGBT) high school students found the percentage of college educated adults in the school district was inversely associated with the frequency of reported verbal and physical harassment and assaults at school, and district-level poverty was positively associated with reported victimisation.^30^ The Urban Men's Health Study, a cross-sectional probability-based sample of men who have sex with men, found socioeconomically deprived sexual minorities reported less social support than their lesser deprived comparators.^31^ These findings are also consistent with the narratives captured in a qualitative study which found working class gay men were hesitant to access support from the gay community.^32^

Our study has some limitations that should be considered when interpreting the findings. First, is the impact of loss to follow-up. We used multiple imputation with sample and attrition weights to maximise the plausibility of the missing at random assumption. Results were comparable when using the datasets with no missing and imputed data, increasing confidence in the findings. Second, as we had relatively small sample sizes, we chose to group people who identified as bisexual, gay or other sexual identities. We also combined young people identifying as bisexual with the larger group who reported they were “mainly heterosexual”. We were unable to determine whether participants sexual identity was disclosed and did not account for, or explicitly model, participants gender identity. There is likely to be variability in the lived experiences of different sexual minority groups, by whether sexuality is disclosed or not, and for people with different intersecting gender and sexual identities, and larger studies should investigate these differences. Third, there is evidence that the validity of self-reported suicide attempts in adolescents may be overestimated due to false positives (eg, incidences without intent to die).^33^ To produce the findings observed here these false attempts would need to vary systematically according to sexuality and socioeconomic deprivation. Fourth, as our analyses were cross sectional and suicide attempt was a lifetime measure, it is possible that attempts occurred before participants were aware of their sexual identity, or at a time when their exposure to socioeconomic disadvantage was different. Fifth, unemployment of resident parents/carers may not completely capture socioeconomic disadvantage if adolescents or their parents/carers have other sources of income. The use of the number of parents/carers unemployed also placed a ceiling on exposure in single parent households. If there was underestimation of exposure or misclassification, this would attenuate associations towards the null. While our sample size was large, in line with much research into interaction we likely had low power to detect an additive–scale interaction, an issue exacerbated by suicide attempts being relatively rare. That we found evidence of a stepped additive interaction between parent/carer unemployment and sexuality suggests the impact is likely to be minimal.

The main finding from this study is that young people who identify as a sexual minority and are socioeconomically deprived are likely to need more support than people with either identity/exposure. Our results underscore the importance of not assuming that the effects of two identities/exposures will be the linear sum of effects. Despite growing up during a period where same sex marriage was legalised, and sexual orientation made a protected characteristic, we observed large inequalities in suicide attempts according to both young people's sexual identity and exposure to socioeconomic deprivation. Based on these findings, there is a need for policies, organizational practices, and school-based interventions to reduce risk factors for suicide and programs to support the mental health of socioeconomically deprived sexual minority young people.

## Contributors

JW was responsible for and led the data analysis, drafting of the manuscript, and literature review. SB and DR substantially contributed to study conceptualisation and drafting of the manuscript. All authors checked the work for intellectual content, read and revised the Article, and approved it for submission.

## Data sharing statement

Data from the Millennium Cohort study is available to researchers upon application (https://discover.ukdataservice.ac.uk/).

## Declaration of interests

We declare no competing interests.

## Appendix A Supplementary data

Supplementary Tables S1–S4

Captions for supplementary material

STROBE_checklist
