# Supplementary material for: Socioeconomic inequalities in the risk of suicide attempts among sexual minority adolescents: Findings from the UK's Millennium Cohort Study
Source: Lancet Reg Health Eur. 2022 Dec 26;26:100570. doi: 10.1016/j.lanepe.2022.100570 (PMC9813783; doi:10.1016/j.lanepe.2022.100570)
Supplement: Supplementary Tables S1–S4 [file mmc1.docx]

**Supplementary information**

1. Table S1. Interaction between living with any unemployed parents and sexual minority status on the risk of suicide attempt (n = 10,247)
2. Table S2. Interaction between housing tenure and sexual minority status on the risk of suicide attempt (n = 10,247)
3. Table S3. Interaction between living with one unemployed parent and sexual minority status on the risk of suicide attempt in participants with no missing data (n = 6,821)
4. Table S4. Interaction between living with two unemployed parents and sexual minority status on the risk of suicide attempt in participants with no missing data (n = 5,372)

Table S1. Interaction between living with any unemployed parents and sexual minority status on the risk of suicide attempt (n = 10,247)

|  | **No unemployed parents/ carers** | **Any unemployed parents/ carers** | **RR (95% CI) for living with Any Unemployed Parents/ carers within strata of Sexual Orientation** |  |
| --- | --- | --- | --- | --- |
|  | **RR (95% CI)** | **RR (95% CI)** |  |  |
| Heterosexual | 1·00 | 1·71 (1·16-2·51) | 1·71 (1·16-2·51) |  |
| Sexual Minority | 2·92 (2·22-3·86) | 4·88 (3·34-7·12) | 1·67 (1·09-2·54) |  |
| RR (95% CI) for Sexual Minority within strata of living with Any Unemployed Parents/ carers | 2·92 (2·22-3·86) | 2·86 (2·13-3·82) |  |  |
| Measure of interaction on additive scale: RERI (95% CI) = 3·85 (0·43-7·27), P = 0·03  Measure of interaction on multiplicative scale: RRs (95% CI) =0·44 (-0·03-0·91), *P* = 0·07  RR: relative risk. CI: confidence interval. | | | | |

Table S2. Interaction between housing tenure and sexual minority status on the risk of suicide attempt (n = 10,247)

|  | **Owned** | **Rented** | **RR (95% CI) for living in a Rented property within strata of Sexual Orientation** |
| --- | --- | --- | --- |
|  | **RR (95% CI)** | **RR (95% CI)** |  |
| Heterosexual | 1·00 | 2·32 (1·62-3·31) | 2·32 (1·62-3·31) |
| Sexual Minority | 2·93 (2·20-3·89) | 6·65 (4·76-9·28) | 1·16 (1·14-1·19) |
| RR (95% CI) for Sexual Minority within strata of living in a Rented property | 2·93 (2·20-3·89) | 2·86 (2·13-3·82) |  |
| Measure of interaction on additive scale: RERI (95% CI) = 2·41 (1·12-3·69), *P <* 0·001 | | | |

Measure of interaction on multiplicative scale: RRs (95% CI) =0·98 (0·65-1.48), *P* = 0·90

RR: relative risk. CI: confidence interval.

Table S3. Interaction between living with one unemployed parent and sexual minority status on the risk of suicide attempt in participants with no missing data (n = 6,821)

|  | **No unemployed parents/ carers** | **One unemployed parent/ carer** | **RR (95% CI) for living with One Unemployed Parent/ carer within strata of Sexual Orientation** |
| --- | --- | --- | --- |
|  | **RR (95% CI)** | **RR (95% CI)** |  |
| Heterosexual | 1·00 | 1·74 (1·19-2·55) | 1·74 (1·19-2·55) |
| Sexual Minority | 2·82 (1·92-4·03) | 4·721 (3·24-6·87) | 1·67 (1·32-2·11) |
| RR (95% CI) for Sexual Minority within strata of living with one Unemployed Parent/ carer | 2·82 (1·92-4·03) | 2·71 (2·11-3·47) |  |
| Measure of interaction on additive scale: RERI (95% CI) = 1·15 (0·55-1·75), *P* = 0·001 | | | |
| Measure of interaction on multiplicative scale: RRs (95% CI) = 0·96 (0·70-1·31), *P* = 0·77  RR: relative risk; CI: confidence interval | | | |
|  | | | |

Table S4. Interaction between living with two unemployed parents and sexual minority status on the risk of suicide attempt in participants with no missing data (n = 5,372)

|  | **No unemployed parents/ carers** | **Two unemployed parents/ carers** | **RR (95% CI) for living with Two Unemployed Parents/ carers within strata of Sexual Orientation** |  |
| --- | --- | --- | --- | --- |
|  | **RR (95% CI)** | **RR (95% CI)** |  |  |
| Heterosexual | 1·00 | 2·00 (1·13-3·53) | 2·00 (1·13-3·53) |  |
| Sexual Minority | 2·82(1·98-4·03) | 8·42 (4·85-14·63) | 2·98 (1·42-6·24) |  |
| RR (95% CI) for Sexual Minority within strata of living with Two Unemployed Parents/ carers | 2·82 (1·98-4·03) | 4·21 (2·88-6·15) |  |  |
| Measure of interaction on additive scale: RERI (95% CI) = 4·60 (0·97-8·22), *P* = 0·01 | | | | |
| Measure of interaction on multiplicative scale: RRs (95% CI) = 1·49 (0·83-2·67), *P* = 0·15  RR: relative risk. CI: confidence interval. | | | | |
