# Supplementary material for: Socioeconomic inequalities in the risk of suicide attempts among sexual minority adolescents: Findings from the UK's Millennium Cohort Study
Source: Lancet Reg Health Eur. 2022 Dec 26;26:100570. doi: 10.1016/j.lanepe.2022.100570 (PMC9813783; doi:10.1016/j.lanepe.2022.100570)
Supplement: Captions for supplementary material [file mmc2.docx]

**Supplementary information**

1. Table S1. Interaction between living with any unemployed parents and sexual minority status on the risk of suicide attempt (n = 10,247)
2. Table S2. Interaction between housing tenure and sexual minority status on the risk of suicide attempt (n = 10,247)
3. Table S3. Interaction between living with one unemployed parent and sexual minority status on the risk of suicide attempt in participants with no missing data (n = 6,821)
4. Table S4. Interaction between living with two unemployed parents and sexual minority status on the risk of suicide attempt in participants with no missing data (n = 5,372)
